# Electrostatic Stabilization of a Native Protein Structure in the Gas Phase**

**DOI:** 10.1002/anie.201005112

**Published:** 2010-11-09

**Authors:** Kathrin Breuker, Sven Brüschweiler, Martin Tollinger

**Keywords:** electron capture dissociation, electrostatic interactions, gas phase, native mass spectrometry, protein structure

Recently, a general picture has been proposed of how long, and to what extent, native protein structure can be retained in the gas phase.1a In particular, molecular dynamics simulations suggest that salt bridges and ionic hydrogen bonds on the protein surface can transiently stabilize the global fold shortly after desolvation.1b However, the use of native mass spectrometry^2^ for studying protein solution structure is still controversial, mostly because site-specific experimental gas-phase data^3^ is scarce. Here we report electron capture dissociation (ECD)^4^ data on the gas-phase structures of the three-helix bundle protein KIX^5^ (Figure 1) that indicate substantial preservation of the native solution structure on a timescale of at least 4 s. We demonstrate that in the gas phase, the most stable regions are those stabilized by salt bridges and ionic hydrogen bonds.

**Figure 1:**
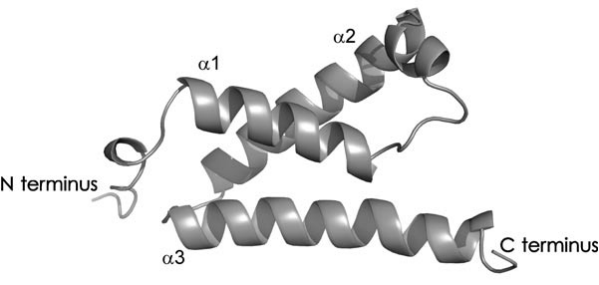
Structure of KIX in aqueous solution at pH 5.5 and 27 °C, as determined by NMR spectroscopic experiments (PDB entry: 2AGH, model 1).^5^

Figure 2 shows site-specific yields of ***c*** and ***z***^•^ fragment ions^6^ from ECD of (M + *n* H)^*n*+^ ions of KIX (see Figure S1 in the Supporting Information) formed by electrospray ionization (ESI).^7^ For the 7+ ions, separated ***c*** and ***z***^•^ products were observed only from backbone cleavage near the termini (residues 1–13 and 89–91), but not from the three-helix bundle region, which forms a globular fold around a hydrophobic core (residues 16–88).^5^ This observation is consistent with intramolecular interactions in the three-helix bundle region preventing separation of ***c*** and ***z***^•^ backbone-cleavage products3a–c in the gaseous 7+ ions. Collisional activation of the 7+ ions (laboratory-frame energy: 28 eV) prior to ECD effected only marginal unfolding near the N terminus (see Figure S2 in the Supporting Information), revealing a notable stability of the three-helix bundle in the absence of solvent.

**Figure 2:**
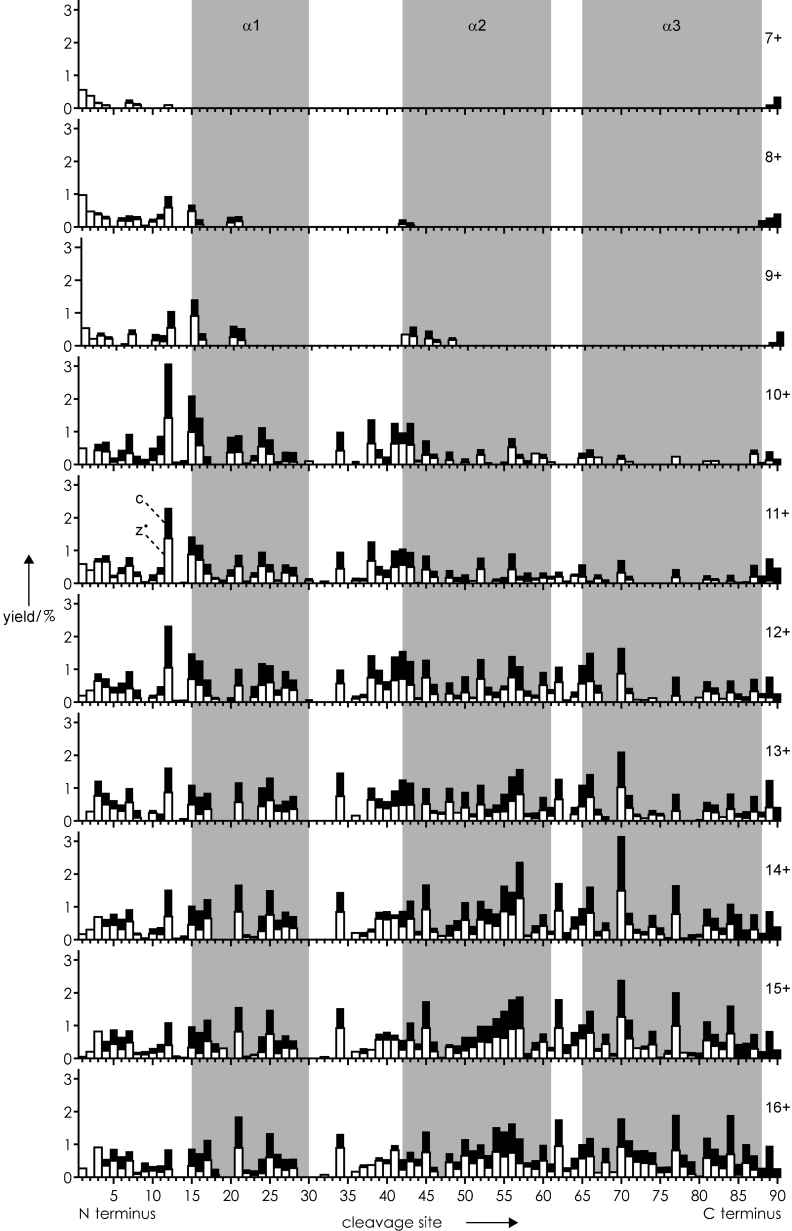
Yields of ***c*** (black bars) and ***z***^•^ (open bars) fragment ions from ECD of (M + *n* H)^*n*+^ ions of KIX versus backbone cleavage site; helix regions are shaded gray. Ions with *n*=7–12 and *n*=13–16 were electrosprayed from quasinative (80:20 H_2_O/CH_3_OH, pH 4) and denaturing (50:50 H_2_O/CH_3_OH, pH 2.5) protein solutions (1–2 μm), respectively.

For the 8+ ions (Figure 2), the appearance of cleavage products from the N-terminal ends of helices α1 (residues 16–30) and α2 (residues 42–61) indicates partial unfolding, with helix α1 separating from the bundle, and helices α1 and α2 starting to unravel from their N-terminal ends. Unraveling of α1 and α2 continues in the 9+ ions, while helices α2 and α3 appear to largely retain their native antiparallel bundle structure. Separation of α2 and unraveling of α3 (residues 65–88), also from its N-terminal end, is evident from the fragmentation pattern observed for the 10+ ions. However, ***c***- and ***z***^•^-ion yields in the 65–88 region remained relatively small for the 10+ and 11+ ions, suggesting that partially intact α3 helix structure limits fragment ion separation. Further increasing the precursor ion charge gave increased ***c***- and ***z***^•^-ion yields and unfolding, similar to ECD data for Ubiquitin3c (see Figure S3 in the Supporting Information), with the fragmentation pattern of the 16+ KIX ions being largely unselective with respect to backbone cleavage site.

The data in Figure 2 provide substantial evidence for a correlation between the solution- and gas-phase structures of KIX. This supposition is corroborated by ECD of 12+ ions generated by nano-ESI from a solution (in H_2_O at pH 4.5) that better resembles the native protein environment,^8^ which gave decreased ***c***- and ***z***^•^-ion yields in the α2 and α3 regions (see Figure S4 in the Supporting Information), along with a smaller total fragment ion yield (37 %) relative to that resulting from ECD of 12+ ions from ESI of solutions in H_2_O/CH_3_OH (80:20) at pH 4 (total fragment-ion yield: 49 %; see Figure S3 in the Supporting Information).

The temporal stability of nativelike KIX 7+ ions was studied by introducing a delay between ion trapping and structural probing by ECD. However, the ECD fragmentation patterns showed no significant differences for delay times of 1 μs and 2 s (see Figure S5 in the Supporting Information). To expedite possible structural transitions, we next activated the gaseous 7+ ions by 28 eV collisions (see Figure S2 in the Supporting Information) prior to ion trapping. Despite the increase in ion internal energy, the fragmentation patterns from ECD with delays of 1 μs, 2 s, and 4 s (Figure 3) are strikingly similar.^9^ Apparently, the three-helix bundle structure of KIX is sufficiently stabilized by specific noncovalent interactions that outweigh the loss of hydrophobic bonding in the gas phase.

**Figure 3:**
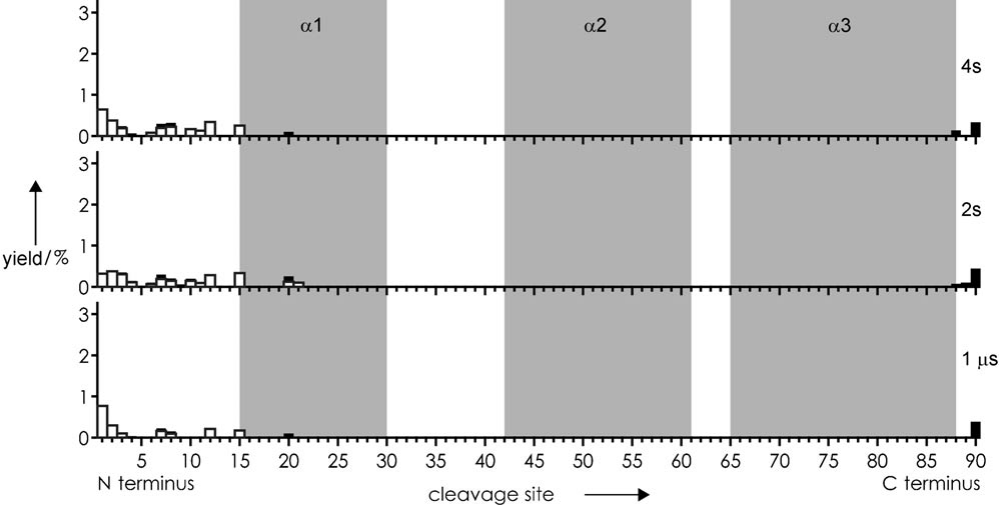
Yields of ***c*** and ***z***^•^ fragment ions from ECD of (M + 7 H)^7+^ ions of KIX electrosprayed from a solution in H_2_O/CH_3_OH (80:20) at pH 4.0 versus backbone cleavage site. The experiments were carried out with collisional ion activation (laboratory-frame energy: 28 eV) and delays between ion trapping and structural probing by ECD of 1 μs (bottom), 2 s (center), and 4 s (top).

Figure 4a shows integrated ***c***- and ***z***^•^-ion yields for helix regions α1, α2, and α3 versus precursor ion charge. The data exhibit sigmoidal behavior, with transition charge values (at 50 % of the plateau value) of 9.2, 10.7, and 12.4 for α1, α2, and α3, respectively. This order of helix stability (α3>α2>α1) in the gas phase agrees with that in solution as determined by NMR spectroscopic experiments.^10^ However, in solution, each helix unfolds cooperatively,^10^ whereas the gas-phase data (Figure 1) show incremental unraveling from their N-terminal ends. This behavior is also reflected in the site-specific transition charge values from analysis of site-specific ***c***- and ***z***^•^-ion yields (see Figure S6 in the Supporting Information), which generally increase from the N to the C terminus (Figure 4b). Transition charge values for cleavage sites between helix regions (31–41, 62–64) are similar to values for adjacent helix ends, indicating that helix separation does not precede helix unraveling.

**Figure 4:**
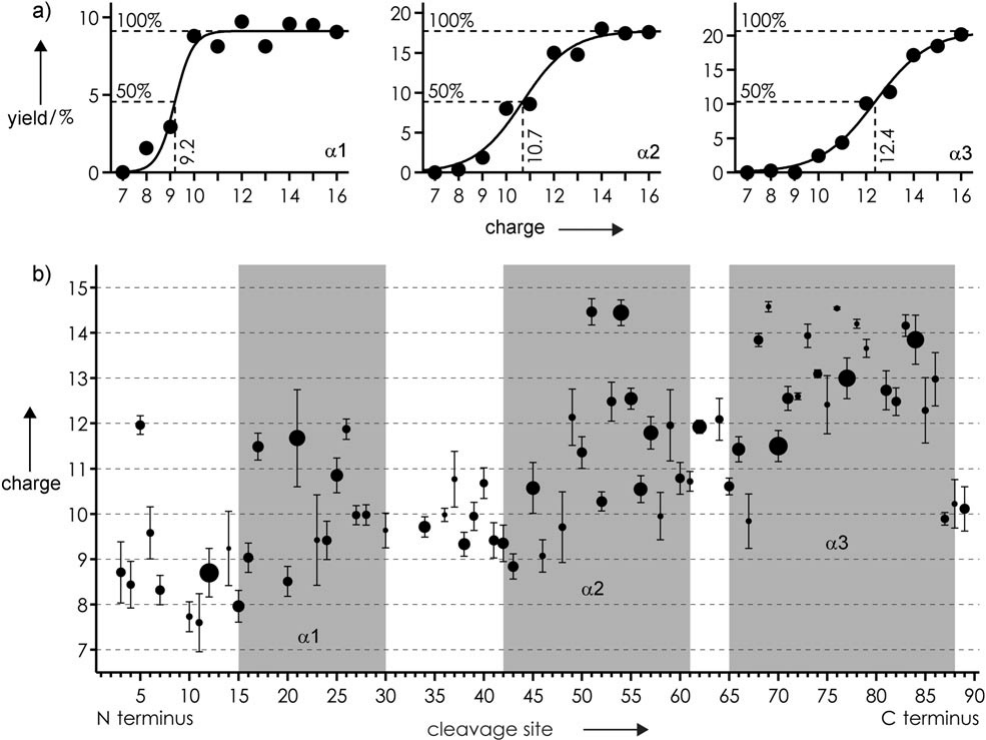
Analysis of the data in Figure 2: a) integrated ***c***- and ***z***^•^-ion yields for helix regions α1, α2, and α3 versus precursor ion charge; b) site-specific transition charge values (at 50 % of plateau value) versus backbone cleavage site; symbol size and error bars represent plateau values and standard deviations for transition charge values from sigmoidal fit functions, respectively.

Although the ECD data in Figures 2 and 3 demonstrate extensive preservation of the native solution structure in the 7+ ions, its stabilization in the gas phase must be based on interactions other than hydrophobic bonding.3d,e These include neutral^11^ and ionic1b, 12 hydrogen bonds, charge–dipole interactions,^13^ and salt bridges.1b, 14 Figure 5 shows helices α1, α2, and α3 with all basic (H, K, R) and acidic (D, E) residues highlighted in color. The density of charged residues is smallest for α1 (5 out of 15 residues, 0.33) and largest for α3 (14 out of 24 residues, 0.58); α2 exhibits an intermediate density of 0.4 (8 out of 20 residues). Importantly, the charge density values correlate (*r*=0.9775) with transition charge values (as a measure of helix stability in the gas phase) for α1, α2, and α3 (Figure 6a). This observation strongly suggests that interactions involving charged residues, that is, ionic hydrogen bonds and salt bridges, largely determine helix stability in the gas phase.

**Figure 5:**
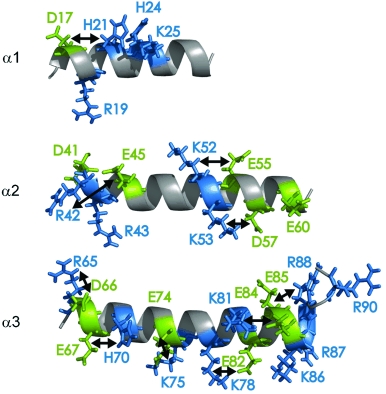
KIX α helices with possible salt bridges between basic (blue) and acidic (green) residues indicated by arrows.

**Figure 6:**
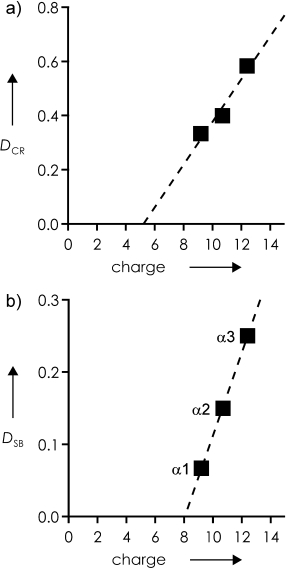
a) Density of charged residues (*D*_CR_, number of charged residues/number of residues) and b) density of salt bridges (*D*_SB_, number of salt bridges/number of residues) versus transition charge value for helices α1, α2, and α3 (linear-fit functions with Pearson correlation coefficients of *r*=0.9775 (a) and *r*=0.9999 (b) shown as dashed lines).

Close inspection of the native KIX structure revealed that one (D17/H21), three (R42/E45, K52/E55, K53/D57), and six (R65/D66, E67/H70, E74/K75, K78/E82, K81/E84, E85/R88) intrahelix salt bridges can stabilize helices α1, α2, and α3, respectively (Figure 5). The density of salt bridges correlates (*r*=0.9999) with transition charge values (Figure 6b) even better than the density of charged residues, suggesting that salt bridges are major determinants for protein structural stabilization in the gas phase. However, this conclusion does not exclude additional stabilization by ionic hydrogen bonds as well as charge–dipole interactions. In particular, interaction of the positive net charge at the C-terminal end of helix α3 (Figure 5) with its electric dipole moment can further stabilize the α3 helix structure,^13^ and is consistent with helix unraveling from the N-terminal end.

Stabilization of the global fold by interactions between the three helices probably involves helix dipole/dipole interactions;^15^ the antiparallel helices α2 and α3 with larger dipole moments than that of the shorter helix α1 separate and unfold last. Additional stabilization of tertiary structure by ionic hydrogen bonding between charged residues and backbone amides1b is indicated by the scatter of site-specific transition charge values (Figure 4b).

We show here that electrostatic interactions can compensate for the loss of hydrophobic bonding and stabilize the native three-helix bundle structure of KIX in the gas phase on a timescale of at least 4 s. Among these interactions, salt bridges were found to play a dominant role. However, a high number of surface-exposed charged residues alone does not guarantee protein stability in the gas phase: equine Cytochrome *c* has 24 basic and 12 acidic residues,3a with the number of salt bridges on the protein surface increasing from 6 in solution to an average value of 17.3 in the gas phase within 10 ps after desolvation,1b yet its native fold disintegrates on a timescale of milliseconds.3e, 16 The outstanding stability of gaseous KIX ions observed in this study must be attributed to the combination of favorable electrostatic interactions, including salt bridges, neutral and ionic hydrogen bonds, as well as charge–dipole interactions. Whether or not native mass spectrometry can reveal information about the solution structure of a protein critically depends on the timescale of the experiment1a and the extent of intramolecular stabilization by electrostatic interactions. KIX is the first protein for which site-specific ECD data indicate preservation of the solution structure in the gas phase. We propose KIX as a model protein for the evaluation of new and emerging methodology for the structural probing of gaseous proteins.

## Experimental Section

KIX protein (91 residues, GSHMGVRKGW HEHVTQDLRS HLVHKLVQAI FPTPDPAALK DRRMENLVAY AKKVEGDMYE SANSRDEYYH LLAEKIYKIQ KELEEKRRSR L) was expressed in *Escherichia coli* cells by using a plasmid that included the CBP KIX coding region^5^ (residues 586–672; residue 586 corresponds to residue 5 in this study) and purified by Ni-affinity and size-exclusion chromatography.^10^ The purified protein was desalted as described previously.^17^ Solution pH was adjusted by addition of acetic acid. Experiments were performed on a 7 T Fourier transform ion cyclotron resonance (FT-ICR) mass spectrometer (Bruker) equipped with an ESI source (flow rate: 1.5 μL min^−1^) and a hollow dispenser cathode operated at 1.6 A for ECD. The desolvation gas temperature was 200 and 150 °C for 80:20 and 50:50 H_2_O/CH_3_OH solutions, respectively. Before ion trapping, precursor isolation (using radiofrequency waveforms), and irradiation with low-energy (<1 eV) electrons for 17–50 ms in the FT-ICR cell, ions were accumulated in the hexapole ion cells for 0.3–2.0 s. Ion activation prior to ECD was realized in the second hexapole by energetic collisions with Ar gas. Between 250 and 500 scans were added for each ECD spectrum. ECD fragment ion yields were calculated as percentage values relative to all ECD products excluding ***a***^•^/***y*** ions,^6^ considering that backbone dissociation of a parent ion gives a pair of complementary ***c*** and ***z***^•^ ions (100 %=0.5 [***c***] + 0.5 [***z***^•^] + [other products], in which other products are reduced molecular ions and products from loss of small neutral species from the latter).3c
