# Supplementary material for: Electrostatic Stabilization of a Native Protein Structure in the Gas Phase
Source: Angew Chem Int Ed Engl. 2010 Nov 9;50(4):873–7. doi: 10.1002/anie.201005112 (PMC3045662; doi:10.1002/anie.201005112)
Supplement: Supplementary file 1 [file anie0050-0873-SD1.pdf]

Angewandte Chemie
Eine Zeitschrift der Gesellschaft Deutscher Chemiker

Supporting Information

© Wiley-VCH 2011

69451 Weinheim, Germany

Electrostatic Stabilization of a Native Protein Structure in the Gas Phase**

Kathrin Breuker, Sven Brüschweiler, and Martin Tollinger*

anie_201005112_sm_miscellaneous_information.pdf

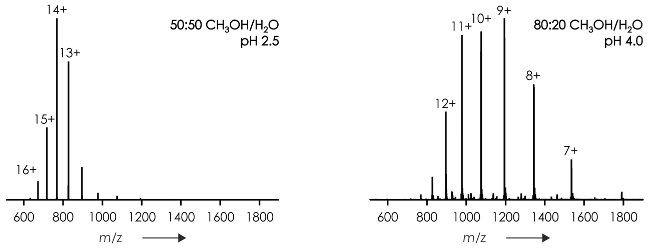

Figure S1: ESI mass spectra of KIX (1-2 μM) electrosprayed from 50:50 H_2O/CH_3OH solution at pH 2.5 (left) and 80:20 H_2O/CH_3OH solution at pH 4.0 (right).

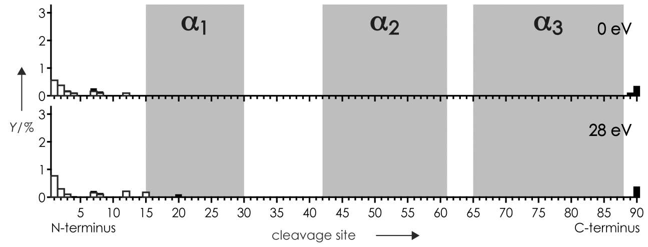

Figure S2: ***c*** (black bars) and $z^{\bullet}$ (open bars) ion yields from ECD of $(M + 7H)^{7+}$ ions of KIX (2 μM) electrosprayed from 80:20 H_2O/CH_3OH solution at pH 4.0 versus backbone cleavage site, without collisional activation (top, which is the same data as in Figure 2) and with 28 eV (laboratory frame energy) collisional activation (bottom) prior to ECD.

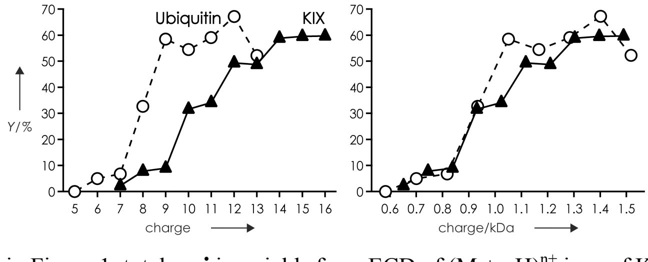

Figure S3: For the data in Figure 1, total ***c***, $z^{\bullet}$ ion yields from ECD of $(M + nH)^{n+}$ ions of KIX (triangles) versus precursor charge (left) and precursor charge divided by protein mass (right); data for Ubiquitin (circles, from reference 3c) are shown for comparison.

SUPPORTING INFORMATION

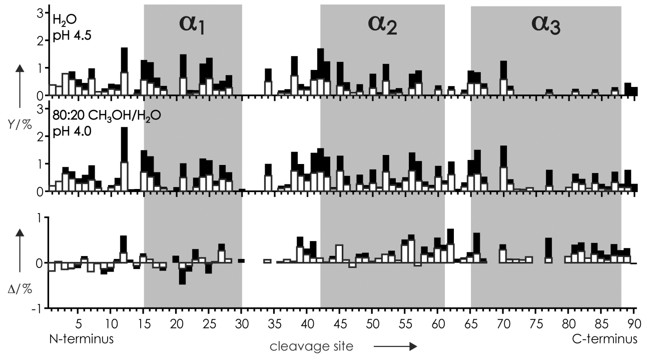

Figure S4: ***c*** (black bars) and ***z•*** (open bars) ion yields from ECD of $(M + 12H)^{12+}$ ions of KIX generated by nano-electrospray ionization from H_2O solution at pH 4.5 (top) and electrospray ionization from 80:20 H_2O/CH_3OH solution at pH 4.0 (center, which is the same data as in Figure 2) versus backbone cleavage site, the bottom trace shows the yield difference.

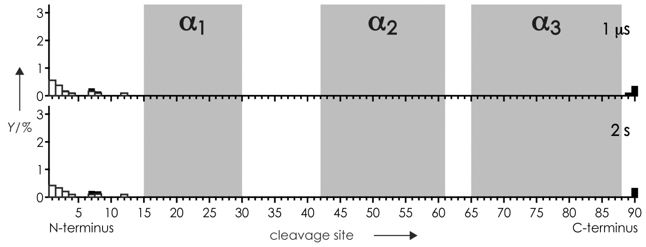

Figure S5: ***c*** (black bars) and ***z•*** (open bars) ion yields from ECD of $(M + 7H)^{7+}$ ions of KIX (2 μM) electrosprayed from 80:20 H_2O/CH_3OH solution at pH 4.0 versus backbone cleavage site, with delay times between ion trapping and ECD of 1 μs (top, which is the same data as in Figure 2) and 2 s (bottom).

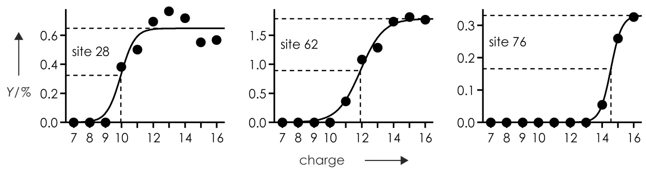

Figure S6: Representative examples for site-specific ***c***, ***z•*** ion yields versus precursor ion charge, solid lines show sigmoidal fit functions from which transition charge values (50% of plateau value) were calculated: 10.0, 11.9, and 14.6 for cleavage sites 28, 62, and 76, respectively.
